# Pre-Damage Strengthening of Heavy-Duty Steel Crane Girders Using Bonded CFRP Plates for Fatigue Life Enhancement

**DOI:** 10.3390/polym18162032

**Published:** 2026-08-21

**Authors:** Xiaoqing Zhao, Yuzhu Liang, Nan Jin, Zhiwei Liu

**Affiliations:** 1 Research Institute of Urbanization and Urban Safety, School of Future Cities, University of Science and Technology Beijing, Beijing 100083, Chinaliuzhiweiim@163.com (Z.L.); 2 Yangtze River Delta Carbon Fiber & Composite Innovation Center, Changzhou 215144, China; 3 Shenzhen Key Laboratory of Urban Disasters Digital Twin, Shenzhen Technology Institute of Urban Public Safety, Shenzhen 518023, China

**Keywords:** steel crane girders, bonding CFRP plates method, thick plate, fatigue life enhancement

## Abstract

In recent years, premature fatigue issues in heavy-duty steel crane girders have occurred frequently, underscoring an urgent need to establish targeted life extension methods. Compared with post-crack repair after macroscopic fatigue cracks have appeared, it is more practical to delay or even prevent the formation of such cracks in fatigue-sensitive zones of the crane girder. Extensive research has demonstrated that bonding Carbon Fiber-Reinforced Polymer (CFRP) plates can significantly enhance the fatigue life of defective components. However, most existing studies focus on thin plates with pre-existing macroscopic cracks, with limited attention given to scenarios involving thick plates or intervention before crack initiation. Therefore, this study focuses on the fatigue problem around bolt holes in the lower flange of heavy-duty steel crane girders. It investigates the life extension method of bonding CFRP plates prior to macroscopic crack formation. Through finite element analysis and comparative fatigue tests, the fatigue life enhancement mechanism was preliminarily interpreted. The effectiveness of this method is validated, and a practical CFRP bonding strategy is proposed to significantly improve the fatigue life of the lower flange in heavy-duty steel crane girders.

## 1. Introduction

**Figure 1:**
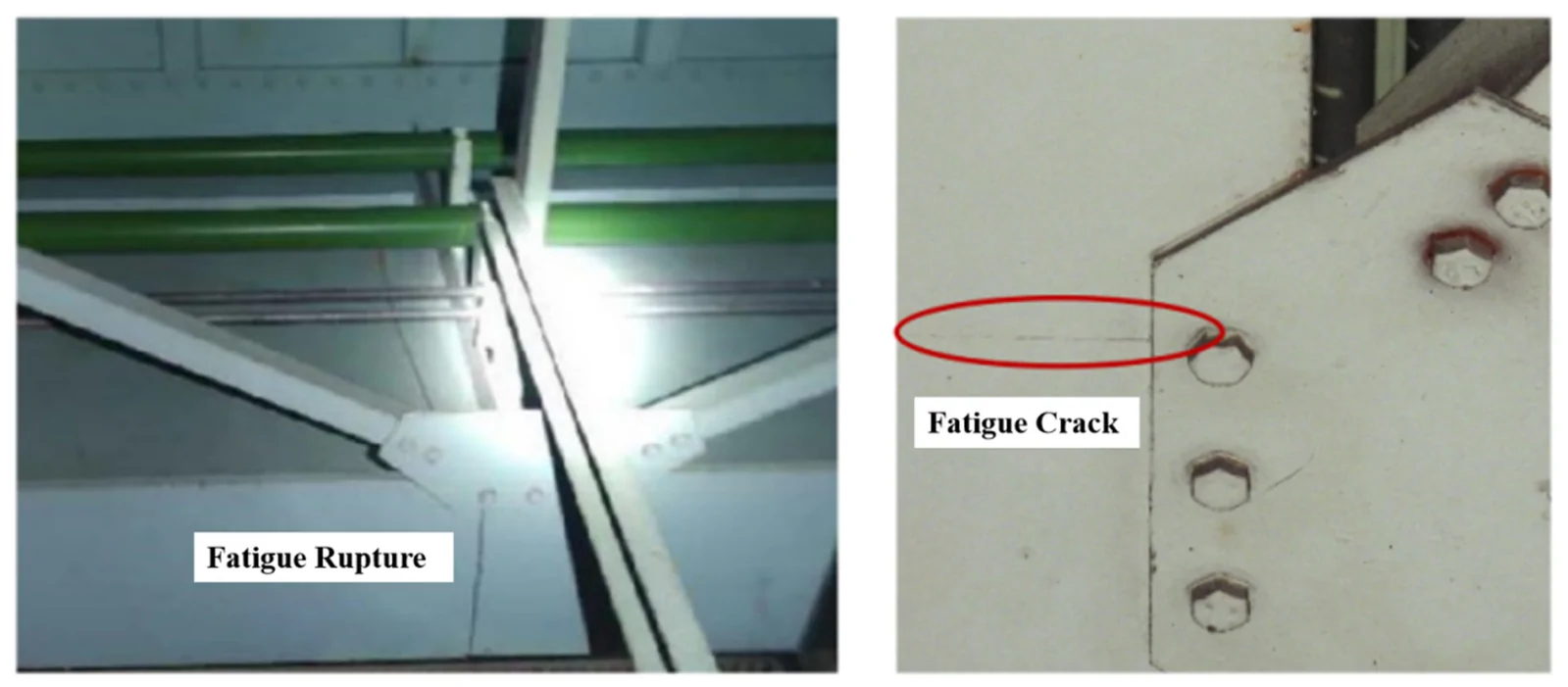
Fatigue fracture at the bolt hole on the lower flange of a steel crane girder.

Steel crane girders constitute critical structural components within heavy industry manufacturing factories. During operation, vertical loads imposed by overhead cranes are primarily supported by the runway beam, while longitudinal braking loads and lateral horizontal loads are sustained by auxiliary structural elements (brake platforms, cross-bracing systems, auxiliary beam, lacing, and horizontal ties) [1,2,3,4,5]. Horizontal ties are typically connected to the lower flanges of the steel crane girders or adjacent to the end of transverse web stiffeners, predominantly utilizing bolted connections. These bolt holes induce localized stress concentrations within the lower flange. However, this stress concentration typically exerts negligible influence on the girder’s overall fatigue strength. Crucially, when the fatigue strength at the critical lower flange-to-web connection satisfies design requirements, the fatigue strength at bolt holes is generally satisfied automatically. Consequently, explicit fatigue verification of bolted connections is routinely omitted in engineering practice. Nevertheless, as numerous industrial structures approach extended service lives, clusters of fatigue-induced failures have recently been documented at these bolted connections in multiple manufacturing factories. Notably, in 2018, fatigue cracking was observed at the bolt holes in the lower flanges of three steel crane girders within a Baotou steel pipe manufacturing facility. As illustrated in Figure 1, identical fatigue cracking was observed circa 2024 in the bolt holes of the lower flanges of five steel crane girders within a Shanghai steel mill. Fatigue fracture occurring within the span of a steel crane girder is recognized as particularly critical, as it can result in crane collapse, rendering it far more consequential than failures in other regions. From a fatigue mechanics perspective, the key scientific issues for such open-hole details involve fatigue crack initiation and subsequent crack propagation under stress concentration, as well as the possibility of reducing the local stress concentration factor through suitable structural strengthening. Given the extensive implementation of this structural configuration throughout heavy industry factories, developing an efficient reinforcement methodology has become imperative.

The lower flange plates of heavy-duty steel crane girders typically have a thickness of approximately 30 mm. Existing strengthening methods primarily aim to enhance load-bearing capacity by increasing the cross-sectional area, with welded strengthening [6] and bolted strengthening [7] being the most common. For fatigue-sensitive bolt hole details, these strengthening approaches may also be understood as practical means to reduce the local stress concentration factor by modifying the sectional stiffness, force transfer path, or local geometry. In practice, welded strengthening dominates. Welded strengthening usually involves attaching an inverted T-section steel member to the lower flange. This shifts the neutral axis of the beam section downward, thereby reducing the stress amplitude in the original lower flange and improving fatigue performance. However, this approach reduces the available headroom and often requires overhead welding during on-site implementation, making quality control difficult and potentially causing secondary damage to the base metal due to heat input. Bolted strengthening typically employs clamping plates connected by friction-type high-strength bolts. This method requires drilling additional bolt holes in the original structure, which further reduces the net cross-sectional area of the lower flange. Furthermore, under long-term dynamic loading, the bolt group is prone to loosening, leading to a loss of preload and a consequent reduction in strengthening effectiveness. To date, no effective method for monitoring preload loss in such bolt groups has been established [8]. Both welded and bolted strengthening techniques exhibit notable limitations. In contrast, CFRP strengthening [9] offers several advantages, including minimal damage to the original structure, low additional weight, and relatively convenient on-site application. Common CFRP strengthening methods for beam-type members mainly include two approaches. The first is the prestressed CFRP method [10,11,12,13,14], which involves tensioning CFRP plates against the lower flange to reduce tensile stress and thereby improve fatigue strength. However, this technique induces tensile stress in the top flange of the crane beam. Since fatigue issues at the connection between the top flange and the web are more challenging to address than those in the lower flange, this method is generally unsuitable. The second is the bonded CFRP method, whose effectiveness largely depends on the adhesive layer and anchorage system. Extensive experimental and field evidence indicates that the primary failure mode of this method is debonding at the plate end. Therefore, CFRP sheets usually need to extend into the zero-moment region and be supplemented with appropriate anchorage measures, such as stainless steel clamp anchors. In addition to strengthening techniques, fatigue monitoring methods may also be used in engineering practice to identify damage development in fatigue-critical details; however, such methods do not directly eliminate stress concentration, and therefore, the present study focuses on strengthening measures rather than structural health monitoring.

Existing research on using bonded CFRP techniques to enhance the fatigue performance of steel structures has predominantly focused on members containing macroscopic cracks. This line of research has mainly addressed cracked steel plates, welded details, and open-hole members with relatively small thicknesses. For instance, studies [15,16,17] investigated steel tension plates (8 mm thick) with 6 mm long central cracks, examining the mechanical mechanism by which bonded CFRP improves fatigue performance. These works proposed a method for calculating the remaining fatigue life after strengthening and introduced a “3D-solid-cohesive-continuous shell” model for the adhesive layer. Another study [18] employed bent steel plates (8 mm thick) with 4 mm long, 2 mm deep semi-elliptical surface cracks at the center. Through comparative fatigue tests, it demonstrated that the bonded CFRP method outperformed bonded steel plate reinforcement in enhancing the fatigue strength of cracked plates under bending. Studies [19,20] explored the effectiveness of bonded CFRP sheets in extending the fatigue life of butt welds using 6 mm thick steel plates with 8 mm long edge cracks. In [21], the mechanism of bidirectional CFRP plates for improving fatigue performance was studied using 2.2 mm thick aluminum alloy plates with central circular holes. Study [22], involving 4 mm thick steel plates with central rectangular holes, proved via comparative fatigue tests that bonded CFRP plates could effectively enhance fatigue life by up to tenfold. A static failure numerical model for the adhesive layer in CFRP applications, based on cohesive zone elements, was proposed in [23], improving the efficiency of finite element modeling and computation. Regarding methods to enhance the fatigue performance of cracked bolt holes, study [24] compared the reliability of stop-hole drilling and bonded CFRP techniques. Results indicated that stop-hole drilling could potentially further reduce the remaining fatigue life, whereas bonded CFRP effectively increased it. The feasibility of using bonded CFRP to strengthen cracked bolt holes was also examined via numerical simulation in [25]. Studies [26,27] utilized high-modulus CFRP sheets, showing that increasing the elastic modulus of CFRP significantly extended the remaining fatigue life of members with cracked bolt holes. An extensive body of research has thus confirmed that bonded CFRP sheets or plates can effectively enhance the fatigue life of thin steel plates with macroscopic cracks. In these studies, plate thickness typically ranged from 8 to 10 mm, and initial crack lengths varied between 2 and 8 mm. Due to the relatively small thickness, the bonded CFRP significantly increased the cross-sectional area of the member, resulting in a notable “bridging effect”. However, the flange plates of heavy-duty steel crane girders generally have a thickness of approximately 30 mm. Given the shear capacity limitations of the adhesive layer, it is challenging to proportionally increase the thickness of the CFRP sheets. Consequently, whether the same strengthening effect can be achieved under thick-plate conditions requires further investigation. Moreover, the majority of a steel member’s total fatigue life—approximately 80%—is consumed before the formation of macroscopic cracks, whereas the remaining life after crack initiation is relatively short. Therefore, extending the fatigue life prior to macroscopic crack formation holds greater practical significance [28,29].

Therefore, for the fatigue problem at bolt holes in the lower flanges of steel crane girders, the bonded CFRP method is considered a suitable strengthening approach. However, existing research on CFRP strengthening for steel structures has primarily focused on thin steel plates with pre-existing cracks—that is, on enhancing fatigue performance after macroscopic cracks have already appeared. Although significant improvements in fatigue performance have been achieved through such strengthening, the remaining fatigue life after crack initiation is still relatively short compared to the life before macroscopic cracking occurs. From an engineering perspective, it is more desirable to substantially extend the fatigue life prior to the appearance of macroscopic cracks. The objective of this study is to evaluate the effectiveness of bonded CFRP plates in improving the fatigue performance of thick-plate bolt hole details prior to macroscopic crack initiation. Accordingly, this study addresses the fatigue issue at bolt holes in the lower flanges of steel crane girders by investigating the bonded CFRP method for enhancing fatigue life before macroscopic crack initiation. More specifically, the present work focuses on pre-damage strengthening of lower flange bolt hole details in heavy-duty steel crane girders rather than on post-crack repair after visible fatigue cracks have already formed. Through finite element analysis and comparative fatigue tests, the effectiveness of the bonded CFRP method is validated, the mechanism by which bonded CFRP plates improve the fatigue life of thick plates without macroscopic cracks is revealed, and a method for calculating fatigue performance after strengthening is proposed.

## 2. Stress State Analysis of Lower Flange Bolt Holes

**Figure 2:**
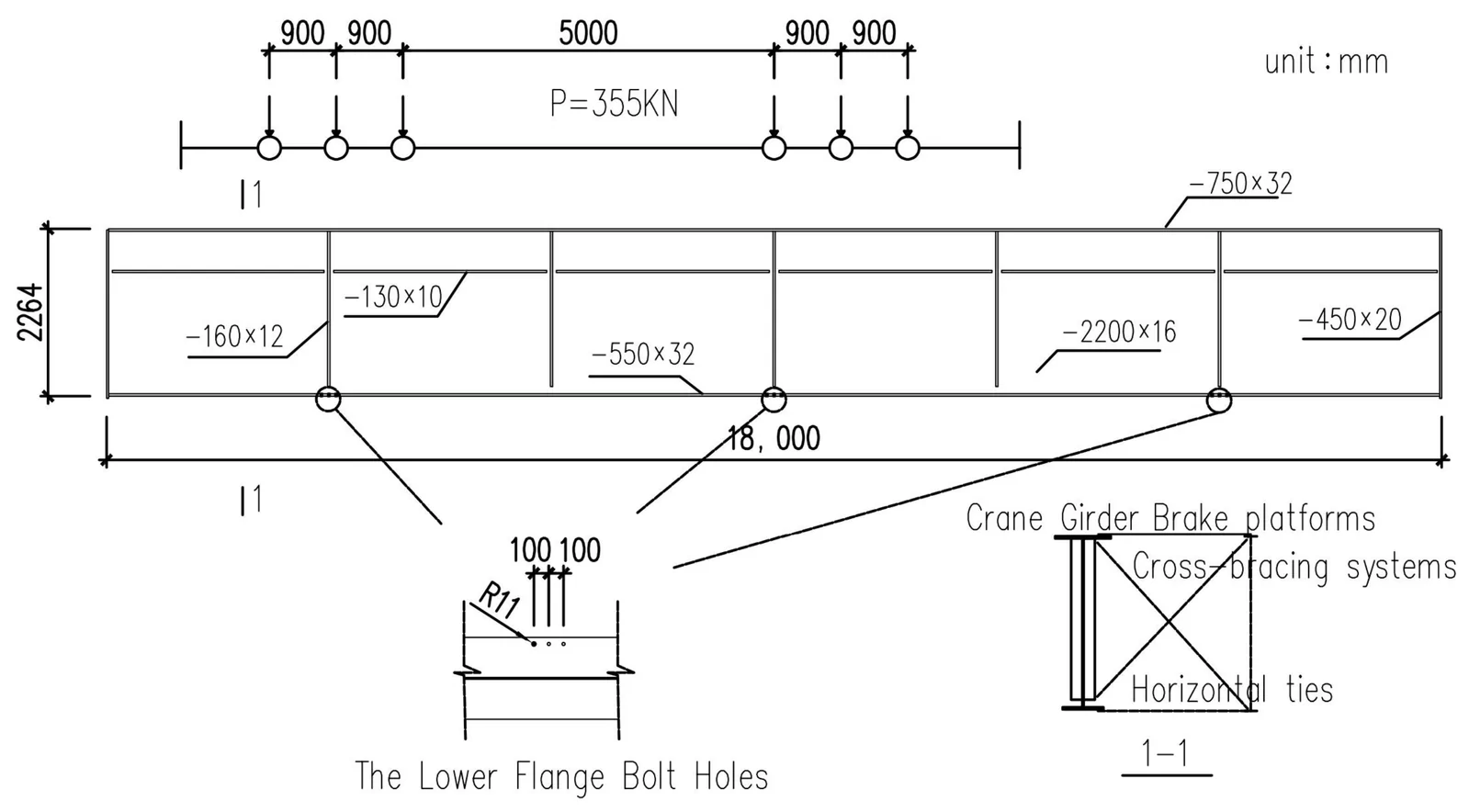
Schematic diagram of the 18 m crane girder system and the most unfavorable load condition.

As depicted in Figure 2, the 18 m span constant cross-section I-girder system represents a standard configuration for heavy-duty crane girder applications. Key structural characteristics include: welded connections between flanges and web plates; braking systems comprising auxiliary trusses and brake platforms; horizontal supports at auxiliary truss lower chords connected to lower flanges via bolting. Material specifications featuring Q345B steel for primary girders and Q235 steel for auxiliary components; top flange-to-web connections employing double-sided complete penetration fillet welds; lower flange-to-web connections utilizing conventional double-sided fillet welds; ordinary bolts specified for auxiliary truss-to-flange connections (though frequently substituted with non-preloaded high-strength bolts in practice). Vertical operational loads from cranes are primarily resisted by the crane girder, representing the principal source of fatigue loading. Fatigue strength calculations typically adopt the load standard value from the most critical crane in the span, considering operational underloading effects. Transverse and longitudinal horizontal loads are collectively resisted by the integrated girder system. Bolt holes connecting horizontal support chords to the crane girder are positioned midway between the span center and the first transverse stiffener at girder ends. Each location features three 22 mm diameter bolt holes with a 100 mm pitch and 45 mm edge distance from flange boundaries.

**Figure 3:**
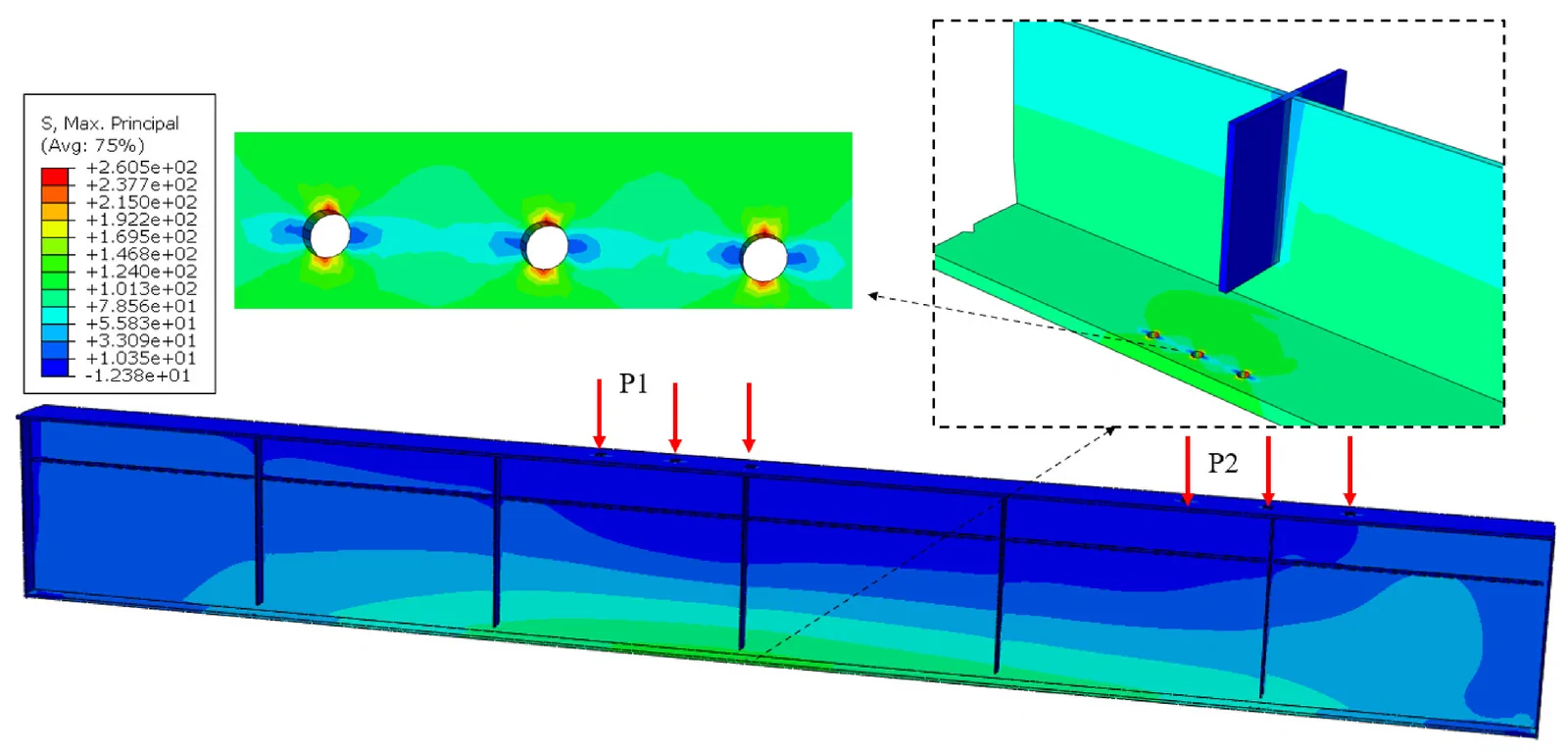
Maximum principal stress of crane girder under the most unfavorable load condition (MPa).

A finite-element model of an 18 m simply supported crane girder was developed using ABAQUS 2021 software. ABAQUS was selected because it provides a reliable platform for three-dimensional stress analysis of complex local details, such as bolt hole stress concentrations, and also allows consistent modeling of the steel–adhesive–CFRP system used in the subsequent strengthened analyses. The loading case producing the most unfavorable stress state at the mid-span bolt hole detail was selected for analysis (Figure 2). Material properties were assigned as linear elastic with an elastic modulus of 2.06 × 10^5^ MPa and Poisson’s ratio of 0.3. All elements were defined as C3D8R. The mesh size at the bolt hole region was refined to 1.5 mm to minimize the influence of mesh discretization on the computational results in the fatigue-sensitive zone, while a coarser size of 20 mm was adopted for other areas. The influence of the brake platforms and auxiliary trusses was neglected in the simulation to simplify the model, as their effect on the local stress state around the selected lower flange bolt hole detail was considered limited. The stress analysis results obtained from the finite element model are presented in Figure 3.

As shown in Figure 3, the maximum principal stress at the bolt hole was calculated to be 350 MPa (stress values at the location remained unchanged with further mesh refinement), while the nominal tensile stress in the net section of the girder’s lower flange was calculated as 121 MPa. This yields a stress concentration coefficient of 2.9 for the bolt hole, consistent with the theoretical coefficient of 3.0 for circular holes in uniaxially loaded plates. Maximum stresses on both the top and lower surfaces adjacent to the bolt hole were found to be nearly equivalent, differing by less than 0.5%. Consequently, the stress state near the bolt hole may be approximated as uniaxial tension.

China’s current *Steel Structure Design Standard* (GB 50017-2017) [24] prescribes a fatigue strength of 112 MPa for this bolt hole configuration, while the European standard BS EN 1993-1-9:2005 [16] specifies 90 MPa. When an underload equivalence factor of 1.0 is applied, compliance is achieved with the Chinese standard but not satisfied under the European provision. Conversely, the nominal tensile stress in the gross section of the girder’s lower flange was calculated as 105 MPa. This value falls below the 112 MPa fatigue strength designated by GB50017-2017 for the lower flange-to-web weld connection. These findings indicate that adequate fatigue performance at bolt holes is generally ensured when crane girder designs incorporate a sufficient R/S (stress ratio) margin. Crane girders failing to meet these requirements should undergo prompt fatigue reinforcement interventions.

## 3. Reinforcement of Bolt Holes with CFRP Plates

**Figure 4:**
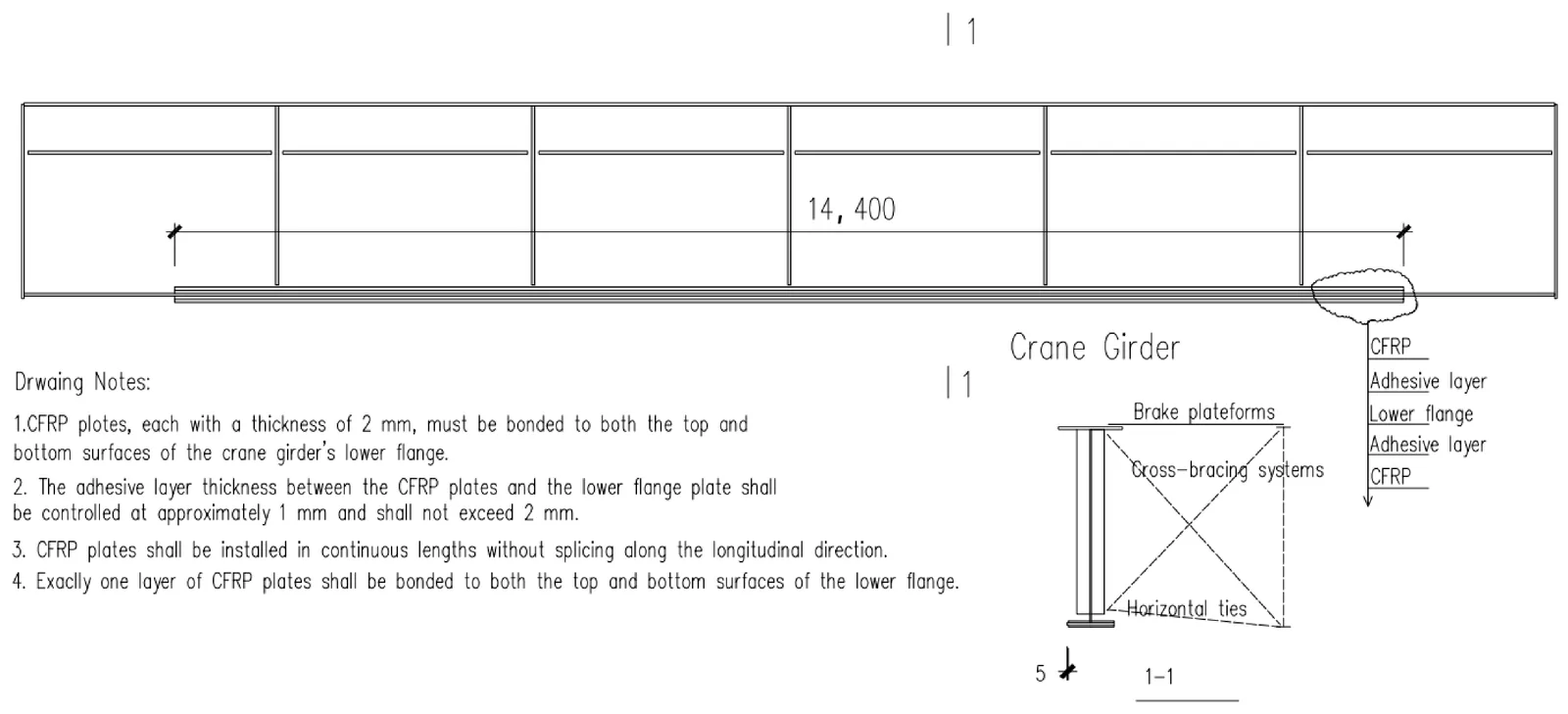
Schematic diagram of the CFRP plate reinforcement method.

As evidenced by the preceding analysis, fatigue failure in these crane girders is attributed primarily to stress concentration at lower flange bolt holes. Consequently, the strengthening scheme should be designed to reduce the local stress concentration at these bolt hole details. The proposed strengthening scheme comprises two key measures: the bolted connections are relocated from the lower flange to the transverse web stiffeners, and the original bolt hole region is strengthened using bonded CFRP plates. Connection of horizontal supports to transverse web stiffeners represents an established construction technique, with reliability demonstrated through extensive engineering practice [26,27,30]. Based on the CFRP strengthening provisions in YB/T 4558–2016 [26], together with the relevant methods reported in Refs. [26,30], a bonded CFRP plate strengthening method is proposed for fatigue-sensitive bolt hole details in the lower flange of steel crane girders, as illustrated in Figure 4.

As illustrated in Figure 4, the CFRP bonding strengthening method described in this paper involves the following procedure. First, the existing connection node between the bottom chord horizontal bracing and the crane girder is relocated to the end of the transverse web stiffeners. Subsequently, CFRP plates are bonded to both the top and lower surfaces of the lower flange. The CFRP plates have a thickness of 2.0 mm, and their width matches the bonding area width on the outer surface of the lower flange plate. On the side without bolt holes, the plate edge is recessed by 5.0 mm to prevent edge tearing of the CFRP due to construction tolerances. The adhesive layer thickness is 1.0 mm. Considering that the crane girder may have undergone long-term service loading, potential fatigue damage invisible to the naked eye may exist around the bolt holes. Therefore, prior to bonding the CFRP plates, the bolt holes should be rehabilitated. This involves polishing the inner walls of the holes using tools such as a grinding wheel and creating a chamfer at the hole edges with a tapered diamond grinding wheel. During CFRP bonding, the bolt holes are filled with resin adhesive. Once cured, this resin exhibits high compressive capacity, which can further mitigate the stress concentration effect around the bolt holes to some extent. Additionally, the six bolt holes on each side of the crane girder also serve as end anchorage points for the CFRP reinforcement.

The feasibility of the bonded CFRP methodology was evaluated using ABAQUS software. Identical load values were applied as described in Section 2. The most unfavorable loading case under single-crane operation was selected, using the standard maximum wheel load. The resin adhesive layer was simulated using a cohesive zone constitutive model, accounting for its static damage during the loading process. The damage evolution law employed the traction–separation law [31], while damage initiation was governed by the quadratic stress criterion. CFRP laminates were assigned a composite laminate constitutive relationship without material failure consideration. Steel components were characterized by linear elastic behavior with failure excluded. Material constitutive relationships are defined as follows:

Steel has a modulus of elasticity of 206 GPa, a Poisson’s ratio of 0.3, and a density of 7.89 × 10^3^ kg/m^3^.

The CFRP plates used in this study have the following properties in the longitudinal direction (0° fiber orientation): tensile strength: 2454 MPa; shear strength: 60.7 MPa; shear modulus: 6 GPa; elastic modulus E1: 168 GPa; elastic modulus E2: 1.1GPa; Poisson’s ratio: 0.25; elongation: 1.66%.

The structural adhesive used in this study had a tensile strength of 46 MPa, a shear strength of 26 MPa, a tensile elastic modulus (Young’s modulus in tension) of 3.2 GPa, a shear modulus of 1.23 GPa, mode I and mode II fracture energy parameters of $G_{I}=0.2$ and $G_{\mathrm{II}}=0.38$, respectively, a Poisson’s ratio of 0.25, and an elongation of 1.5%. It should be noted that the value of 3.2 GPa (3200 MPa) corresponds to the tensile elastic modulus of the adhesive and is therefore higher than the requirement of 2500 MPa, whereas 1.23 GPa refers to the shear modulus rather than the tensile modulus.

**Figure 5:**
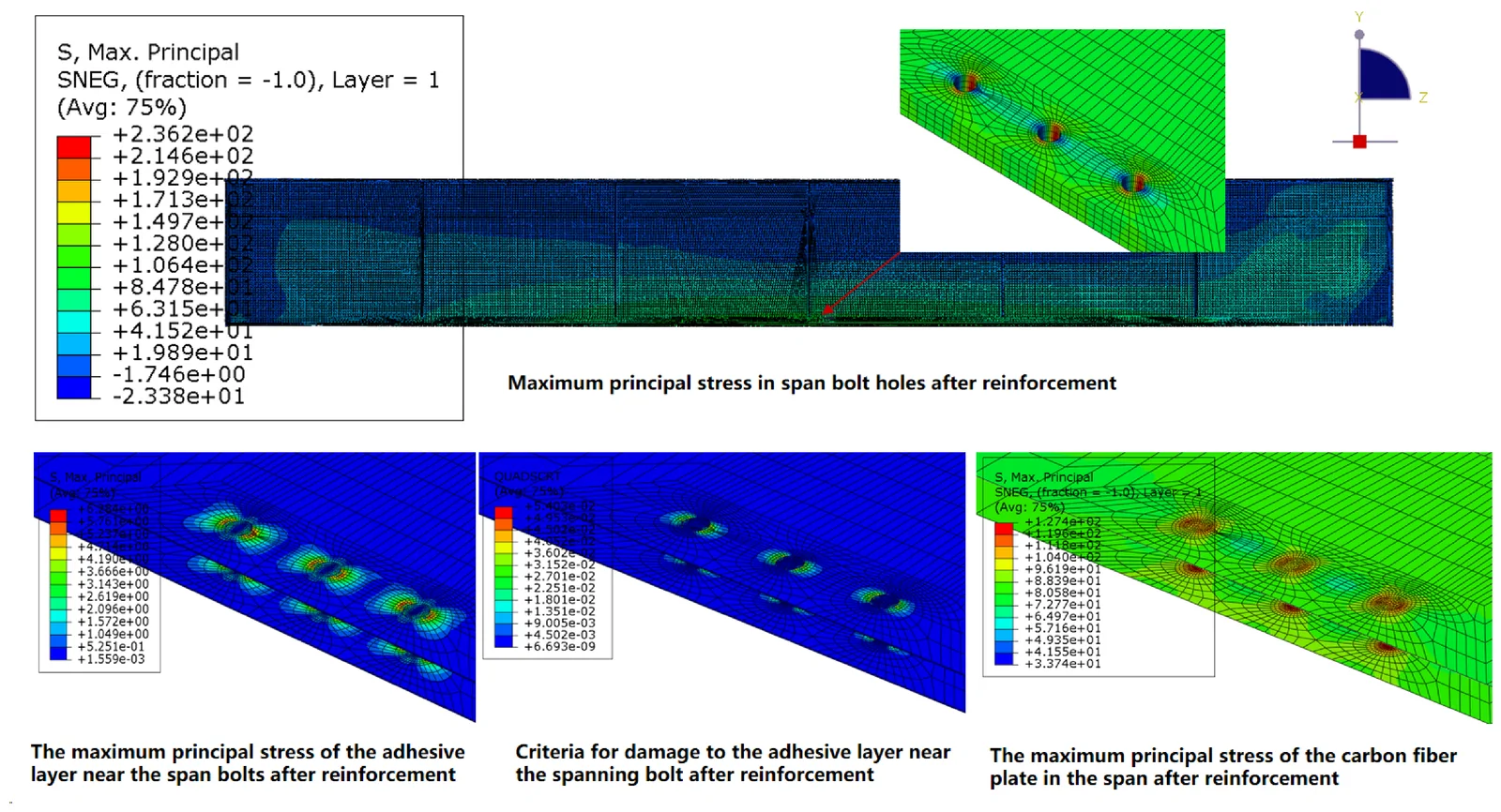
Stress distribution near the mid-span bolt hole after CFRP reinforcement (MPa).

As presented in Figure 5, the maximum principal stress at the span bolt holes was calculated to be 236 MPa following implementation of the proposed reinforcement scheme. The maximum principal stress in the structural adhesive was 6.3 MPa, and the corresponding damage variable was 0.054. This damage value is significantly lower than the critical threshold of 1.0. The CFRP plate demonstrates a maximum principal stress of 127.4 MPa.

**Figure 6:**
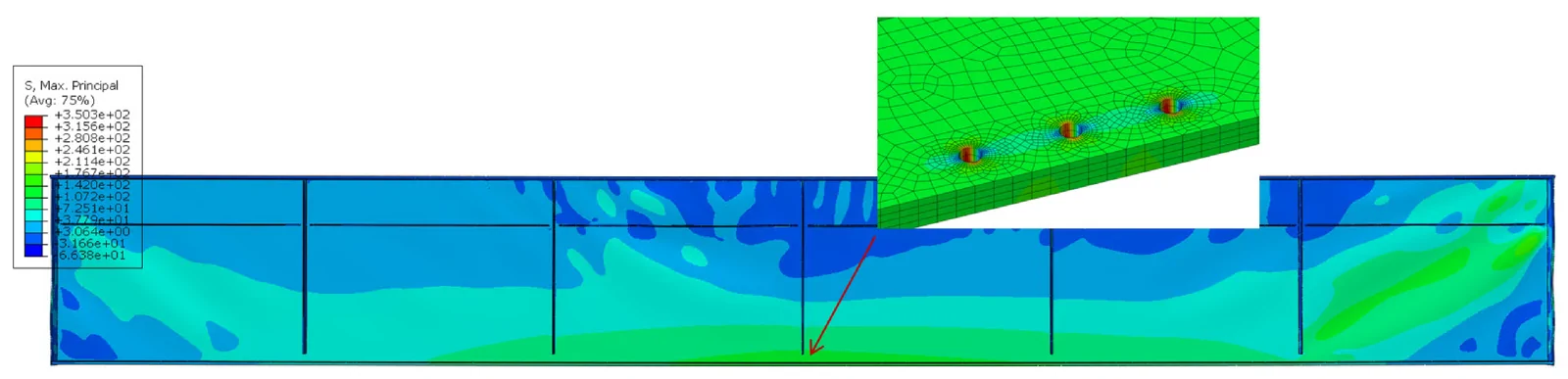
Maximum principal stress nephogram of the prototype under the same mesh division (MPa).

To facilitate a direct comparison of stress reduction, the prototype was analyzed using the same mesh scheme and stress contour scale. The results are shown in Figure 6.

As illustrated in Figure 6, the maximum principal stress near the prototype bolt holes was calculated to be 350 MPa. Following implementation of the proposed CFRP reinforcement scheme, a 32% stress reduction at span-center bolt holes was achieved relative to the prototype configuration (Figure 5), calculated as: (Prototype value − Reinforced value)/Prototype value.

These results demonstrate that the proposed bonded CFRP strengthening scheme is effective in reducing the stress concentration around the bolt hole detail, significantly mitigating stress concentration around bolt holes. When CFRP plates extend to girder ends adjacent to the first transverse web stiffener, additional anchorage measures are unnecessary. Static load validation confirms adequate bearing capacity compliance. According to fatigue verification provisions in YB/T4558-2016 [27], stress levels in reinforced regions should be reduced for fatigue strength calculations. Based on empirical evidence in structural engineering practice, a 30% stress reduction typically corresponds to approximately double the fatigue life.

## 4. Comparative Fatigue Tests

### 4.1. Pilot Program

**Figure 7:**
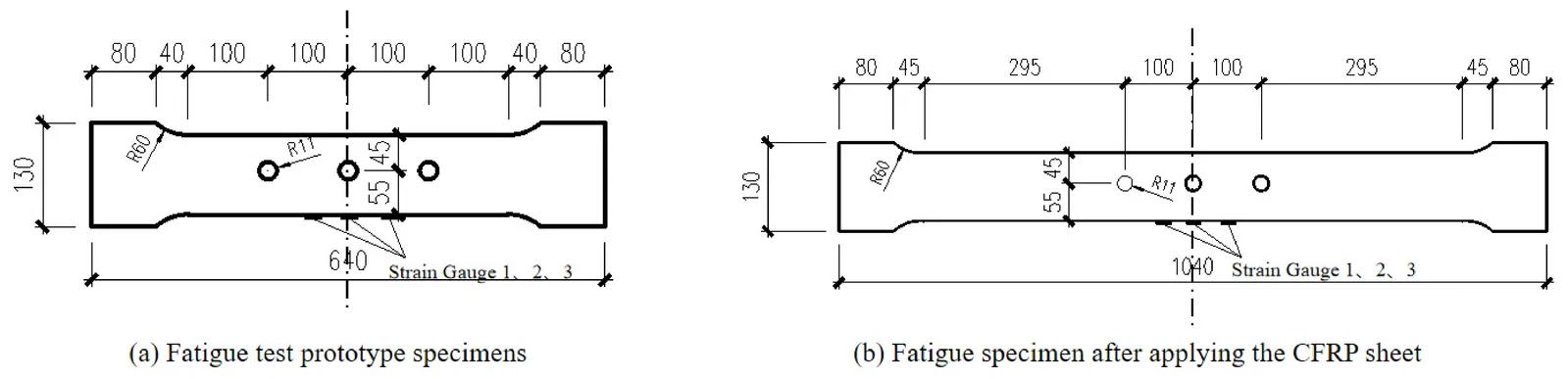
Fatigue test specimens.

To validate the fatigue performance enhancement achieved by the proposed bonded CFRP strengthening method, three groups of comparative fatigue tests were conducted. Specimens were designed according to stress distribution similitude principles (Figure 7a). An unreinforced control group was prepared using identical specimens subjected to the same surface treatment required for CFRP bonding, so that the influence of surface preparation on fatigue behavior could be evaluated separately. Finally, CFRP-reinforced specimens were prepared, as shown in Figure 7b. Three specimen groups were fabricated, each containing two identical units. All specimens featured 32 mm plate thickness matching the crane girder’s lower flange dimensions. Three 22 mm diameter bolt holes were machined centrally via cold drilling, replicating the girder’s hole pattern. The CFRP bonding protocol comprised the following steps:

(i) Steel surface treatment

Scribing: Use an oil-based marker to draw a line where the CFRP plates will be placed. This line will be used to position the paste at a later stage.

Sanding: First, use a steel wire wheel to sand the steel surface in the area where the CFRP plates will be pasted to remove rust, scale, and oxides. Then, use 240-grit sandpaper to partially sand the steel surface to remove stubborn oil stains or oxides.

Sandblasting: Place the test piece in the sandblasting equipment and spray 0.1 mm aluminum oxide onto the surface of the steel plate to ensure relatively uniform sandblasting treatment.

Cleaning: Use alcohol-soaked, dust-free paper to wipe the paste area and remove particles from the test piece. Leave it to dry.

(ii) Surface processing of the CFRP plates:

Use 240-grit sandpaper to sand the adhesive surface of the CFRP plates using a 45-degree cross-sand method to remove the surface texture. Since the CFRP plates tend to stick to the surface of the thicker glue layer during the demolding process, it is important to minimize the glue layer to protect the bonding strength. The sanding process should be even to avoid excessive sanding, which can damage the surface fibers. Generally, the surface of the CFRP plates is dull after polishing. After sanding, use dust-free paper dipped in alcohol to wipe the paste area and remove particles from the CFRP plates. Then, air dry.

(iii) Bonding the CFRP plates.

The CFRP plates used in this study were CARBON CFP carbon-fiber plates manufactured by Carbon Technology Group Co., Ltd. (Tianjin, China), with a nominal thickness of 2.0 mm. The bonding adhesive was a two-component structural epoxy adhesive developed in collaboration with Carbon Technology Group Co., Ltd. for steel–CFRP strengthening applications. As this adhesive is a co-developed product that has not yet been publicly commercialized, the exact commercial designation is not given here. The adhesive was selected because it is suitable for room-temperature field application, compatible with the adopted steel–CFRP strengthening system, and capable of providing adequate stiffness and bond strength for stress transfer in the strengthened detail. The main properties of the CFRP plates and the adhesive used in this study are summarized in Table 1 and Table 2, respectively.

The CFRP plates should be pasted as soon as possible after completing the previous two steps. If more than 24 h elapse, a new rust layer will form on the surface of the steel plate. The relevant requirements of the pasting process are as follows:

Thickness control: Place 1 mm thick pads at both ends of the bonding area to control the thickness of the adhesive layer to about 1 mm.

Formulation of adhesives: Prepare adhesives A and B for the test piece according to the specified ratio. Mix the two components at low speed in one direction to produce fewer bubbles. The mixing time is about three minutes.

Adhesive application: Adhesive is uniformly distributed across the CFRP laminate surface using a notched trowel, maintaining 1–2 mm thickness. The pre-coated laminate is positioned onto the prepared steel substrate. Bond line thickness is controlled to approximately 1 mm using calibrated steel shims. This bond-line thickness was selected to maintain a relatively uniform interfacial stiffness and load-transfer condition, while avoiding an excessively thick adhesive layer that could reduce joint stiffness and aggravate local peel/shear effects near the plate ends. Uniform pressure is applied through calibrated weights or mechanical clamping systems, inducing controlled adhesive squeeze-out. Excess adhesive is removed immediately using solvent-saturated lint-free wipes.

Curing: Use a weight of about 0.5 kg to press both ends of the steel substrate, ensuring the adhesive is 1 mm thick. Observe every 8 h to see if the CFRP plate has shifted until the adhesive is initially cured (about 2 days).

Using the same construction technique, pull-out specimens were fabricated with 40 mm × 40 mm square CFRP plates bonded to the steel substrate. Three pull-out tests were conducted, yielding ultimate failure loads of 10.0 kN, 8.2 kN, and 7.2 kN, respectively. All measured values significantly exceed the code-specified requirement of 4 kN. These results confirm adequate bond quality under the adopted construction procedure and also indicate that the selected adhesive system can provide sufficient interfacial resistance for the present strengthening configuration.

**Figure 8:**
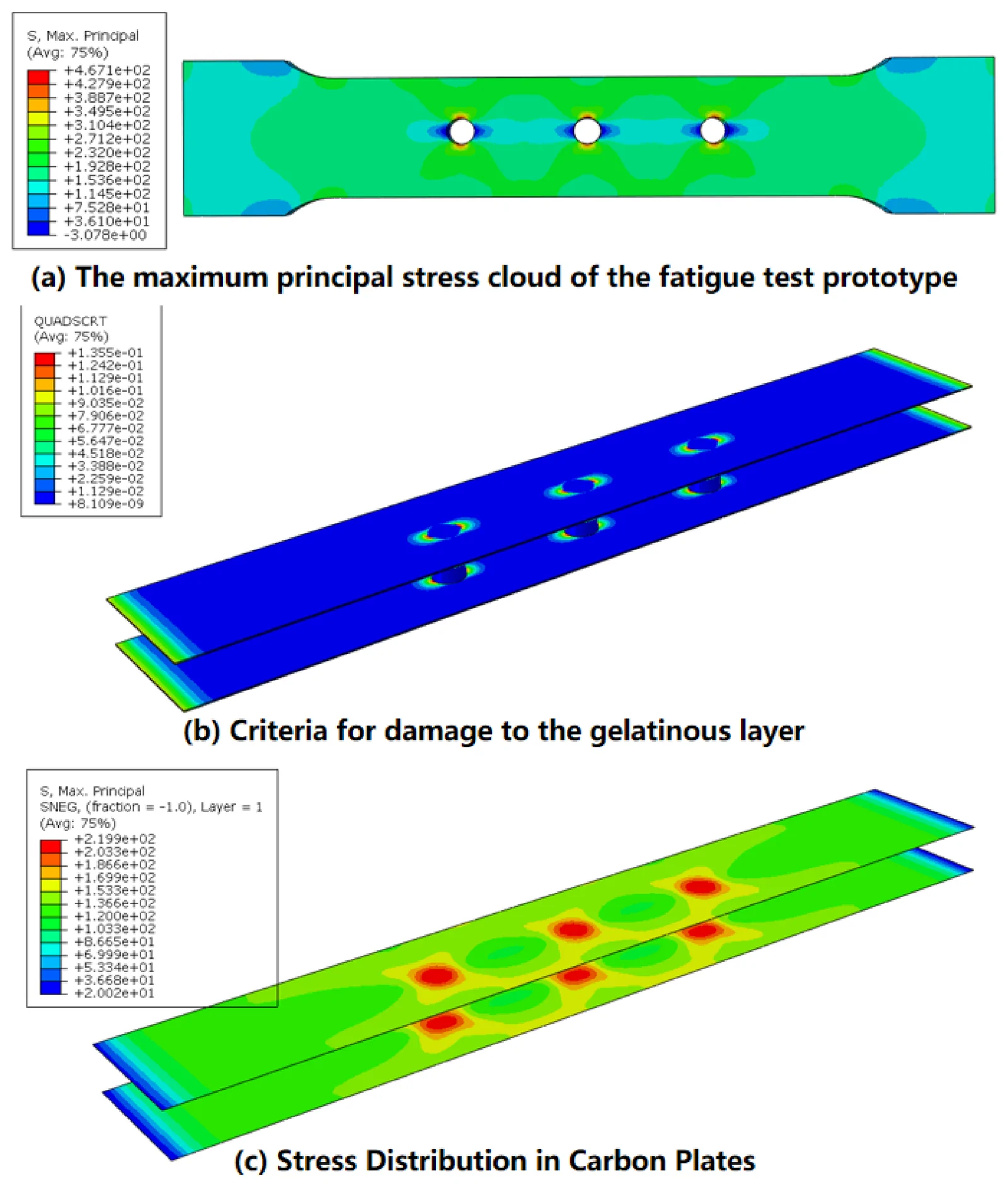
The finite element calculation results of fatigue test specimens (MPa).

Finite element models of both prototype and CFRP-reinforced specimens were developed using the methodology established in Section 3. A tensile load of 568 kN was applied in simulations. Resultant computational data are presented in Figure 8a–c. Comparative analysis of Figure 3 and Figure 8a confirms that stress distributions in the prototype specimen under axial tension satisfy similitude principles with crane girder bolt hole configurations. Figure 8b indicates that no obvious adhesive-layer delamination was predicted under the applied static loading condition. This result suggests that, under the present static loading level, the selected adhesive, the controlled bond-line thickness, and the adopted plate extension length provided adequate interfacial stiffness for load transfer. For fatigue-strengthened members, however, joint performance depends not only on bond strength but also on adhesive stiffness, stress redistribution along the bonded region, and the concentration of peel and shear stresses near the plate ends. This interpretation is consistent with general design principles for bonded joints, according to which joint strength and long-term reliability should be improved by maintaining adequate adhesive-layer stiffness, controlling bond-line thickness, and reducing end stress concentrations through proper joint geometry and anchorage design [32]. Therefore, in the present strengthening scheme, the CFRP plates were extended toward the low-moment/end region, and the repaired bolt hole/end region was utilized to improve load transfer and reduce the risk of premature end debonding. As shown in Figure 8c, the bonded CFRP plates help alleviate the local stress concentration around the bolt hole region through load sharing and stress redistribution.

### 4.2. Test Results

The three groups of specimens were loaded using a 100 t MTS fatigue testing machine. An industrial camera recorded the specimens’ failure process during loading, and a dynamic strain collector recorded the stress state near the bolt holes. Strain gauges were set up as shown in Figure 7a. The upper fatigue load limit was set to 568 kN, the lower load limit to 56.8 kN, and the nominal stress amplitude, calculated on the basis of the net section and excluding the contribution of the CFRP plate and resin adhesive, was 204 MPa. The stress ratio R was set to 0.1, and the loading frequency to 5–8 Hz. The specimen elongated 18 mm when the loading condition terminated. The strain-gauge data recorded during cyclic loading were further converted into local stress–cycle histories for the selected monitoring points, and the representative results are presented in the following subsections.

**Figure 9:**
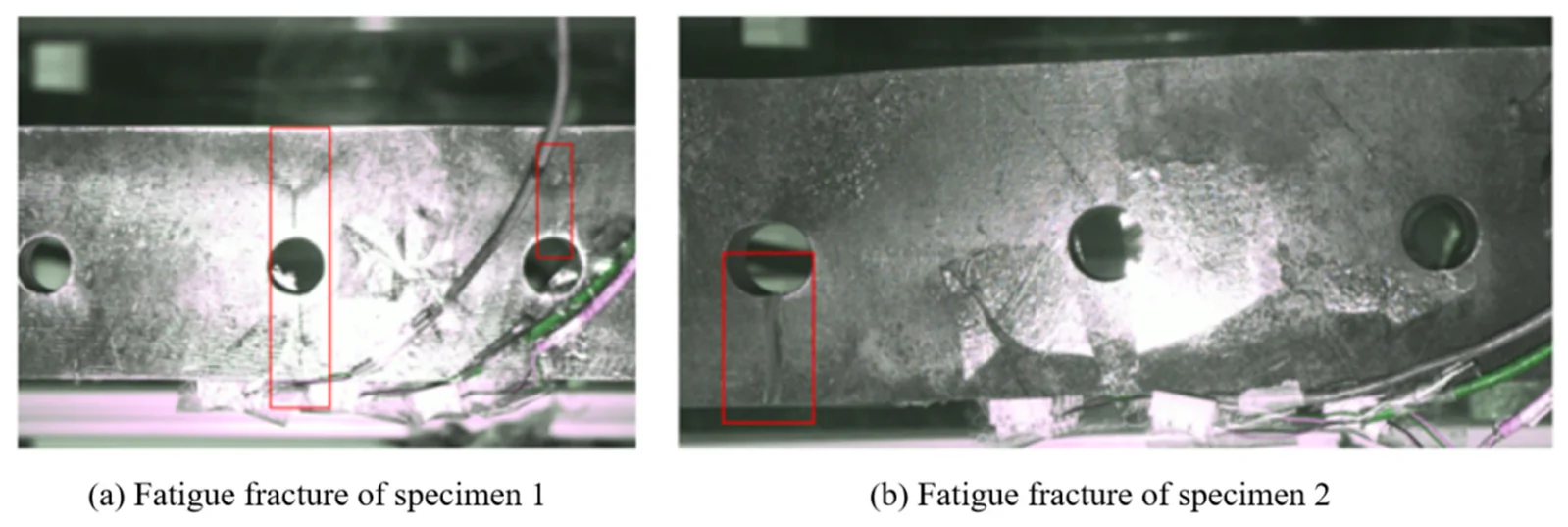
Fatigue test results of prototype specimens.

(i) Test results of prototype specimens:

Specimen 1 was subjected to 250,000 loading cycles, culminating in fatigue fracture under the specified loading regime. Crack initiation was first observed at the minor axis of the central bolt hole, followed by crack propagation at the major axis. Subsequent crack formation was documented at the major-axis edge bolt hole shortly after central hole failure. During final failure stages, necking phenomena were evident at the minor-axis edge bolt hole, as illustrated in Figure 9a.

Prototype Specimen 2 was subjected to 316,000 loading cycles under the specified fatigue regime prior to fatigue fracture. Crack initiation was first observed at the minor axis of the edge bolt hole, followed by necking development at the major axis. No fatigue crack formation was detected at remaining bolt hole locations, as documented in Figure 9b.

**Figure 10:**
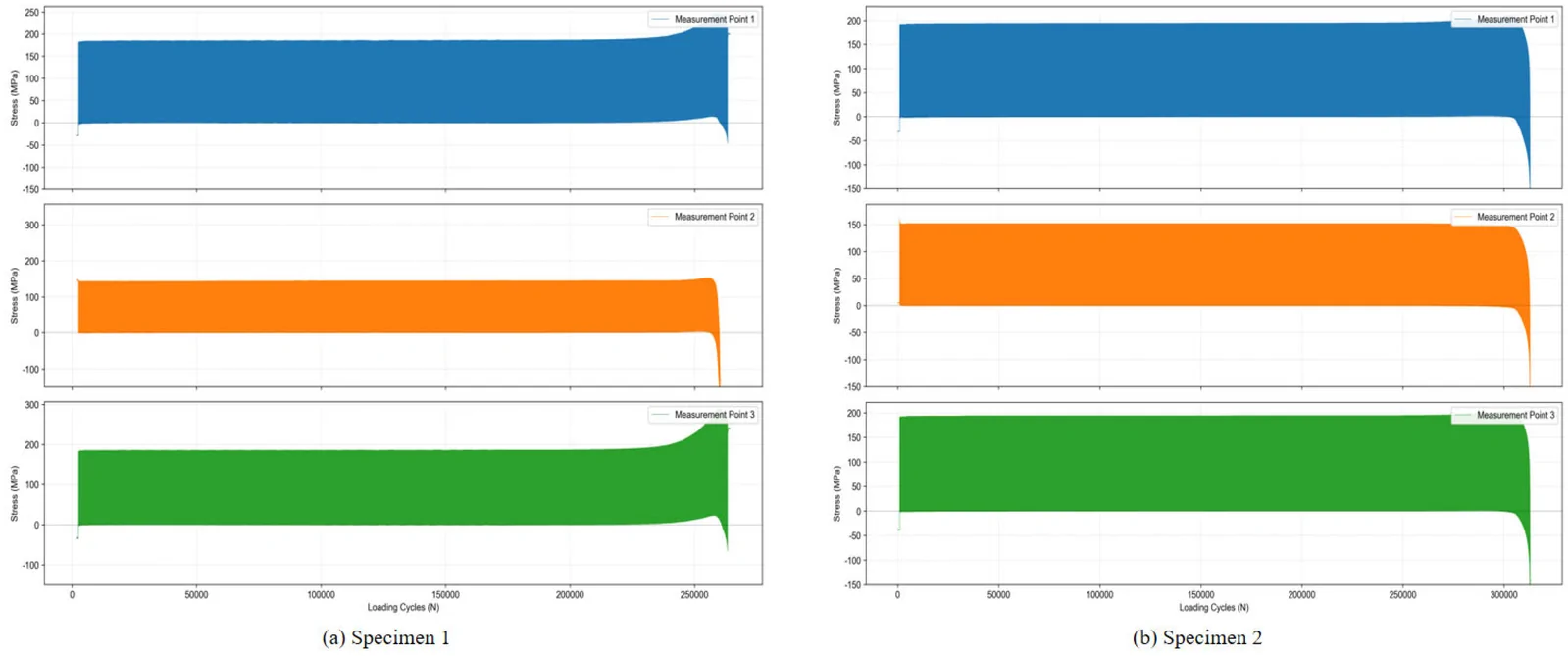
Evolution of local stress at three monitoring points during cyclic loading for Prototype Specimens 1 and 2.

To further illustrate the local stress response during cyclic loading, the stress–cycle histories recorded at the monitoring points of Prototype Specimens 1 and 2 are presented in Figure 10a and Figure 10b, respectively. For both specimens, the stress responses at the three monitoring points remained generally stable during most of the loading process, indicating that the local stress state near the bolt hole region was relatively steady before significant fatigue damage developed. In the final stage of loading, noticeable changes occurred in the recorded stress histories, including a local increase in stress at some monitoring points followed by a rapid drop, which is consistent with the initiation and propagation of fatigue cracks and the final loss of load-carrying capacity.

(ii) Test results of surface-treated specimen

**Figure 11:**
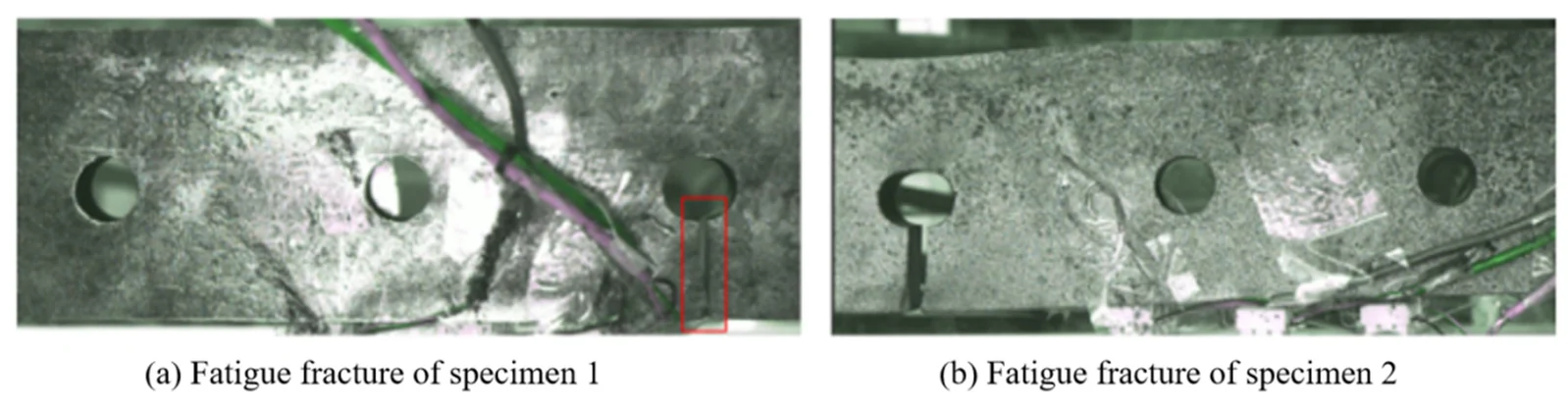
Fatigue test results of the specimen after passing through the surface.

Specimen 1 was surface-treated and loaded 540,000 times under the above fatigue load, resulting in fatigue. Cracks appeared on the narrow side of the edge bolt holes during the loading process, though no fatigue cracks appeared in other positions. Necking appeared on the wide side of the edge bolt holes in the late stage of failure, as shown in Figure 11a.

Specimen 2, which was surface-treated, was loaded 316,000 times under the aforementioned fatigue load and subsequently fatigued and fractured. Cracks appeared on the narrow side of the bolt hole at the edge of the specimen during loading, but no fatigue cracks appeared in other positions. Necking occurred on the wide side of the bolt hole at the edge during the final stage of failure, as shown in Figure 11b.

**Figure 12:**
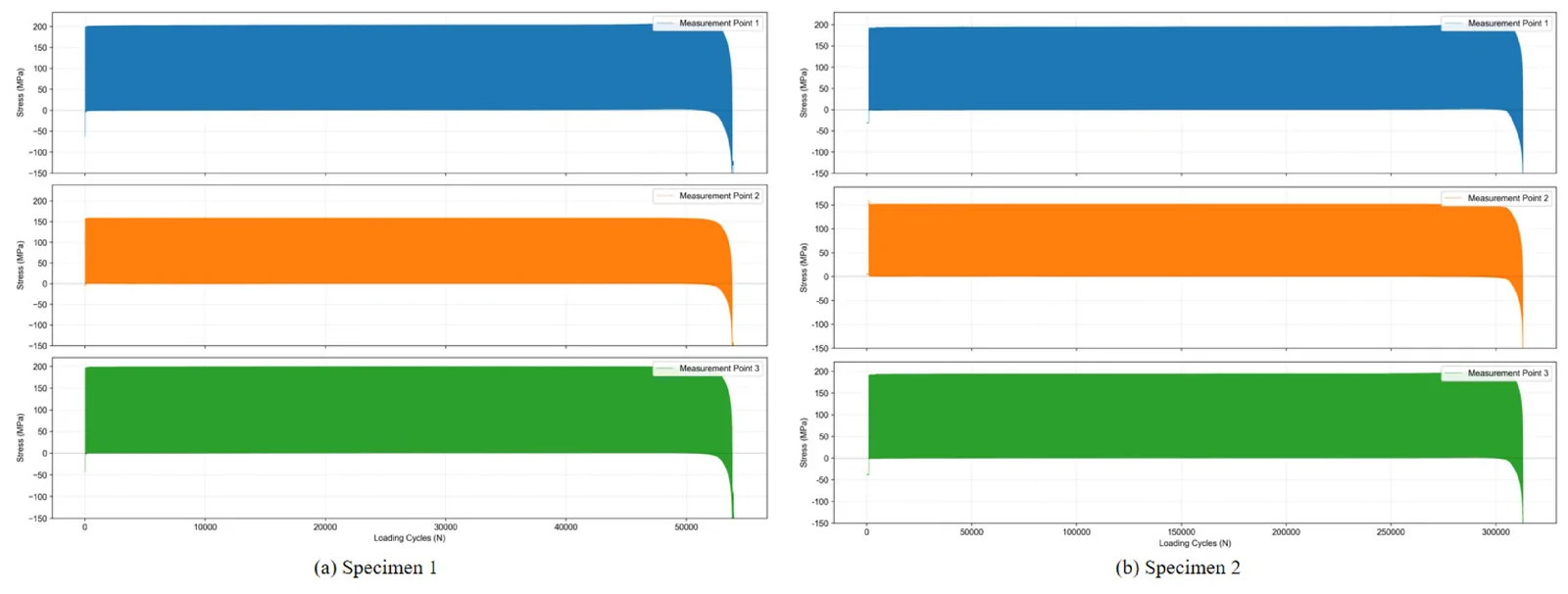
Evolution of local stress at three monitoring points during cyclic loading for surface-treated Specimens 1 and 2.

**Figure 13:**
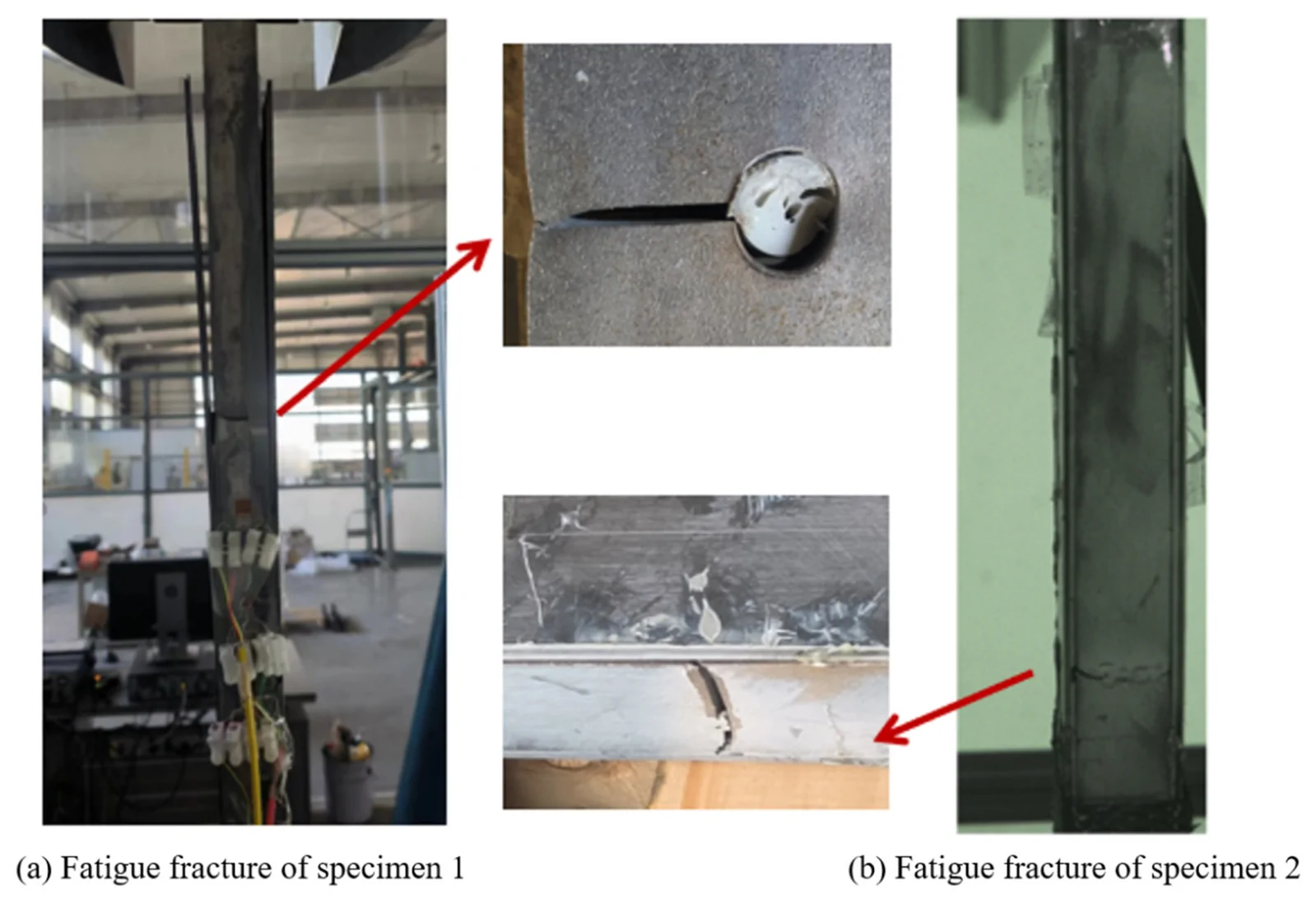
Fatigue test results of specimens pasted with CFRP.

The stress–cycle curves of the monitoring points for specimens during the loading process are shown in the Figure 12. The stress–cycle curves of the specimens after sandblasting treatment are basically consistent with those of the prototype specimens. Sandblasting treatment only prolonged the fatigue crack initiation or nucleation time, while having no effect on the subsequent rapid failure stage.

(iii) Test results for the specimen after the CFRP plates was adhered:

After CFRP plate adhesion, Specimen 1 was subjected to 820,000 loading cycles under the prescribed fatigue loading condition, after which fatigue fracture occurred. From the start of loading to 72,900 cycles, local cracking was observed on the CFRP plate bonding side. Between 72,900 and 141,000 cycles, partial cracking occurred at one end of the adhesive anchoring area of the specimen. By 323,000 loading cycles, the cracked region of the adhesive layer had further expanded toward the bolt holes. No cracking was found in the anchoring bonding area of the other specimen. At 586,000 cycles, the adhesive layer of the specimen was almost completely cracked, with similar cracking observed on the opposite adhesive layer side. While no obvious damage was detected on the alternate adhesive side, the adhesive layer on one side of the specimen exhibited near-complete cracking. At 826,000 loading cycles, fatigue fracture was observed in one of the debonded-side bolt holes, prompting termination of loading, as shown in Figure 13a.

For Specimen 2, fatigue fracture occurred after 960,000 loading cycles following CFRP plate attachment. At 85,000 cycles, loosening of the carbon fiber cloth in the CFRP plate anchoring area was observed. By 360,000 cycles, the degree of loosening had increased, with no cracking or deglossing of the adhesive layer noted in other areas. The same phenomenon was observed at 586,000 cycles as at 360,000 cycles. When loading reached 960,000 cycles, fracture was detected at the edges of the specimen’s bolt holes, leading to loading termination, as illustrated in Figure 13b.

**Figure 14:**
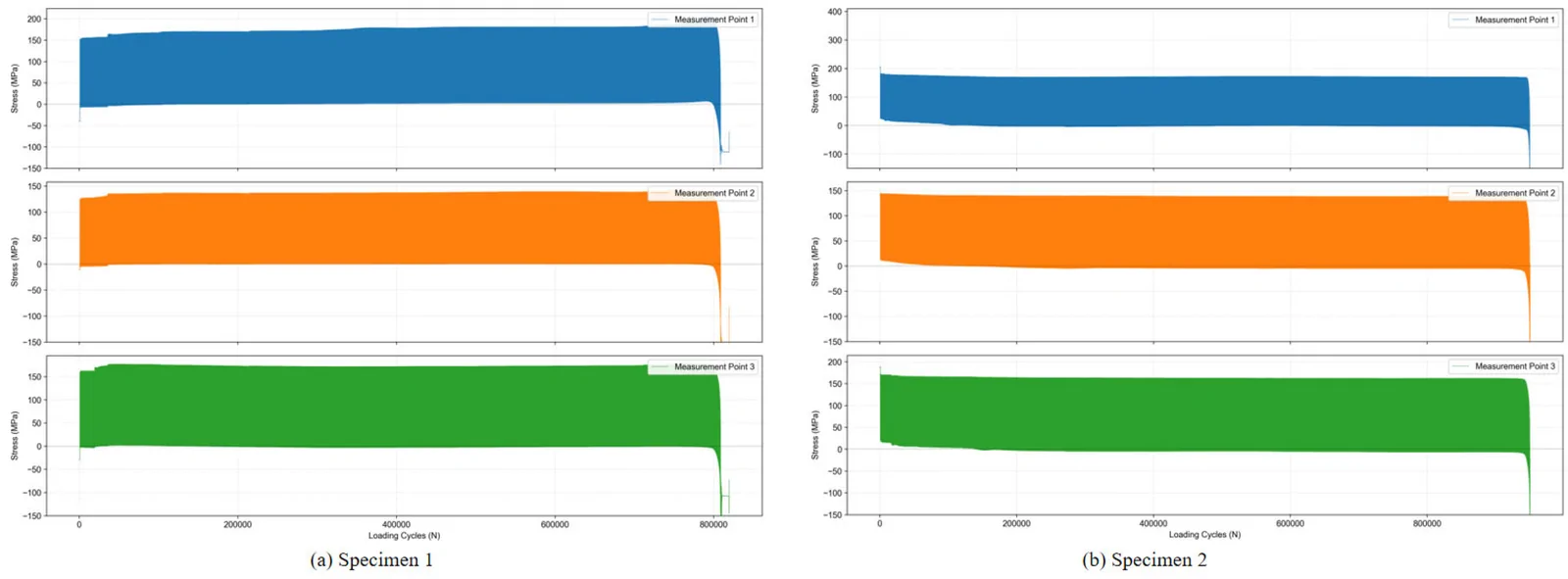
Evolution of local stress at three monitoring points during cyclic loading for CFRP-strengthened Specimens 1 and 2.

The stress–cycle curves of the monitoring points for specimens during the loading process are shown in the Figure 14. The phenomena or variation trends observed from these stress–cycle curves are similar to those of the sandblasted specimens, with no particularly significant differences. This further indicates that CFRP plate strengthening extends the fatigue life primarily during the crack initiation stage, while having limited influence on the rapid failure period.

**Figure 15:**
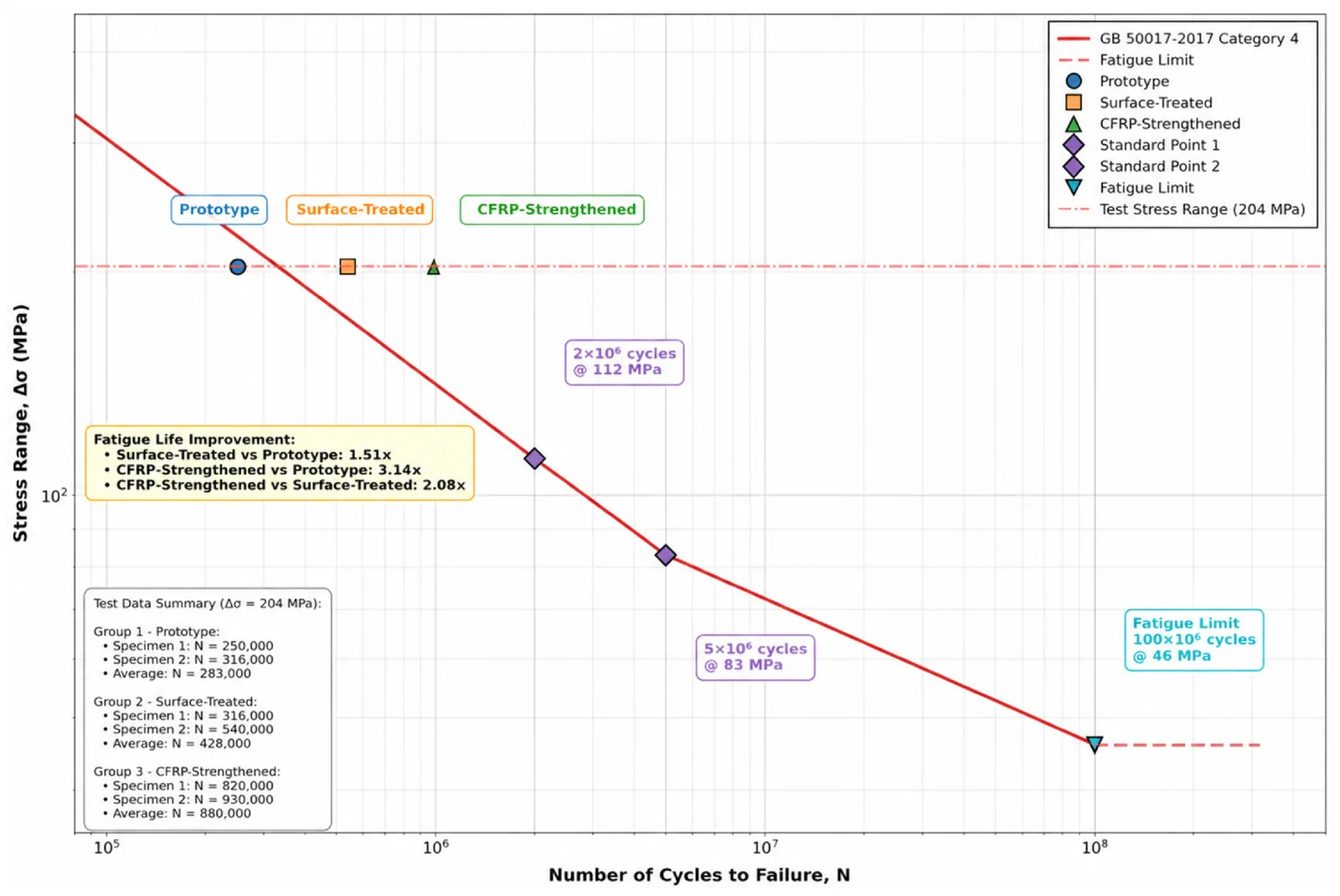
Comparison of fatigue test data with code-specified S-N curve for bolt holes.

The three groups of fatigue test data obtained in this study are compared with the S-N curve for bolt holes specified in the current steel structure design code, as summarized in Figure 15. As can be clearly observed from Figure 15, the bonded CFRP plate method proposed in this study significantly improved the fatigue strength of bolt holes in thick steel plates. Specifically, the fatigue life of CFRP-strengthened specimens was approximately 3.14 times that of the prototype specimens under the same stress range of 204 MPa.

## 5. Discussion and Analysis

Fatigue failure in the lower flanges of steel crane girders is primarily attributed to stress concentration surrounding bolt holes. As established in Section 2, a stress concentration coefficient of approximately 2.9 was determined for these bolt holes, which is slightly lower than the theoretical value of 3.0 for a circular hole in an infinite plate under uniaxial tension, likely due to the finite plate geometry and boundary effects. This deviation is attributed to the proximity of bolt holes to plate edges, resulting in reduced boundary constraints.

In China’s current Standard for Steel Structure Design Standard (GB50017-2017 [24]), the fatigue strength of crane beams’ lower flange-to-web connection welds and that of bolt holes in the lower flange base material are both specified as 112 MPa. In the fatigue strength calculation, the nominal stress of the gross cross-section is adopted for the lower flange and web connection welds, whereas the nominal stress of the net cross-section is used for bolt holes. The difference between these two values is typically negligible, such that satisfaction of the former’s fatigue strength calculation inherently satisfies the latter. The recommended fatigue strength for lower flange-to-web connection welds in the European standard BS EN 1993-1-9:2005 [16] aligns with China’s current standard. However, the recommended value for bolt holes is comparatively lower at 90 MPa. Based on the fatigue test results documented in Section 4 of China’s standard and BS EN 1993-1-9:2005 [16], the fatigue lives of the first two test groups are 327,000 and 1.7 million cycles, respectively. Comparative analysis of the fatigue test results indicates that the fatigue life predictions of China’s standard are conservative: three-quarters of the specimen lives are below the calculated values. In contrast, the recommended values in BS EN 1993-1-9:2005 [16] demonstrate better rationality.

Comparative fatigue testing of three specimen groups indicates that surface preparation prior to CFRP plate bonding may enhance bolt hole fatigue strength marginally, though such improvement is characterized by inconsistent results. This variability is attributed to thickness-related challenges in achieving uniform surface treatments (e.g., grit blasting) across bolt hole surfaces. CFRP plate application was demonstrated to increase specimen fatigue life by approximately 200% relative to unreinforced prototypes. Notably, this significant life extension was achieved despite only an 18% reduction in stress amplitude, indicating that the fatigue life calculation methodology specified in YB/T4558–2016 may be considered conservative. The reinforcement technique simultaneously reduces member stresses and modifies structural details.

The observed damage evolution, together with the numerical results in Section 3 and the pull-out test results, suggests that the repaired bolt hole region can provide a favorable anchorage condition for the CFRP plates under the present configuration.

## 6. Conclusions

A bonded CFRP methodology for mitigating fatigue in heavy-duty steel crane girder lower flange bolt holes is presented in this study. The reinforcement efficacy was validated through finite element analysis, comparative fatigue testing, and supporting analytical work. Principal findings are summarized as follows:

1. Fatigue failure in the lower flange of this crane girder type is primarily attributed to stress concentration at bolt holes. Improved reliability in fatigue strength calculations is demonstrated when BS EN 1993-1-9:2005 provisions are implemented for these structural details.
2. The bonded CFRP methodology effectively enhances fatigue strength of these critical details. An 18% stress amplitude reduction is achieved, while fatigue life exhibits a substantial extension. This life extension significantly exceeds the local stress reduction effect, confirming CFRP’s efficacy in enhancing fatigue performance of thick-plate components prior to macroscopic crack initiation.
3. For field applications, existing beam-end bolt holes are utilized effectively for CFRP anchorage, eliminating the requirement for supplemental end-anchorage measures.

## Figures and Tables

**Table 1:** Some mechanical performance indexes of Carbon Fiber-Reinforced Polymer (CFRP) plates of carbon.

Performance Program	Material Grade	Performance Indicators
Standard value of tensile strength	Class I	≥2400 MPa
Modulus of elasticity in tension	Class I	≥160 GPa
Elongation	Class I	≥1.6%
Fiber volume content	Class I	≥65%
Interlayer shear strength	Class I	≥50 MPa
Tensile bond strength between fiber composite and base material		For steel substrates: ≥3.5 MPa, and shall not be destroyed by adhesion

**Table 2:** Some mechanical performance indexes of the resin adhesive of Carbon Fiber-Reinforced Polymer (CFRP) plates of carbon.

Performance Program		Performance Indicators	Performance Program		Performance Indicators
Colloidal properties	Tensile strength	≥38 MPa	Bonding ability	Steel-to-steel tensile shear strength	Standard value ≥ 14 MPa
	Modulus of elasticity in tension	≥2500 MPa			Average value ≥ 16 MPa
	Elongation	≥1.5%		Steel-to-steel bond tensile strength	≥40 MPa
	Compressive strength	≥70 MPa		Steel-to-steel T impact stripping length	≤20 mm
	Bending strength	≥50 MPa, and shall not be broken in pieces		Positive tensile bond strength of steel to C45 concrete	≥2.5 MPa, and for concrete cohesion damage
Heat deflection temperature (B method using 0.45 MPa bending stress) ≥65 °C			Non-volatile matter content (105 ± 2 °C, 180 ± 5 min) ≥99%		

## Data Availability

The original contributions presented in this study are included in the article. Further inquiries can be directed to the corresponding author.
